# Antisense Oligonucleotide-Mediated *XIST* Knockdown Modulates Endogenous Pluripotency-Associated Transcription Factor Expression in Porcine Female Somatic Cells

**DOI:** 10.3390/ani16182881

**Published:** 2026-09-13

**Authors:** Xiaoyu Chen, Xiaohong Chu, Jing Huang, Liang Zhang, Fuzeng Lu, Nana Yang, Lihe Dai, Ruhai Xu

**Affiliations:** 1 Zhejiang Key Laboratory of Livestock and Poultry Biotech Breeding, Institute of Animal Husbandry and Veterinary Sciences, Zhejiang Academy of Agricultural Sciences, Hangzhou 310021, China; cxy_v@163.com (X.C.); zhuxh@zaas.ac.cn (X.C.); lufuzeng@zaas.ac.cn (F.L.); yangnana0815@163.com (N.Y.); 2 Institute of Virology and Biotechnology, Zhejiang Academy of Agriculture Science, Hangzhou 310021, China; huangjing@zaas.ac.cn (J.H.); hnszhangliang@163.com (L.Z.)

**Keywords:** *XIST* knockdown, antisense oligonucleotides (ASOs), pluripotency-associated transcription factors (pTFs), porcine female somatic cells, cell reprogramming

## Abstract

The long non-coding RNA *XIST* is a key regulator of X-chromosome inactivation (XCI) and contributes to epigenetic regulation during cell reprogramming. However, whether *XIST* suppression influences endogenous pluripotency-associated transcriptional programs in porcine somatic cells remains unclear. In this study, we investigated the effects of *XIST* knockdown on endogenous pluripotency-associated transcription factors (pTFs) in IBRS-2 cells. Chemically modified antisense oligonucleotides (ASOs) targeting *XIST* were designed and transfected into IBRS-2 cells. *XIST* expression was efficiently suppressed for up to 120 h after ASO treatment. RT-qPCR analysis showed that *XIST* knockdown induced sustained upregulation of *KLF4* and LIF expression, prolonged intermittent activation of *NANOG*, and transient activation of *G6PD*. In contrast, *OCT4*, *SOX2*, and *c-MYC* were significantly upregulated only during the early stage after transfection. KLF4 immunofluorescence was detectable in a subset of ASO-2-treated cells. These findings demonstrate that ASO-mediated *XIST* knockdown partially facilitates the activation of a subset of endogenous pluripotency-associated genes in porcine female somatic cells. This study provides new insights into the role of *XIST* as an epigenetic regulator during porcine somatic reprogramming and suggests that optimized *XIST* interference may represent a potential strategy for enhancing endogenous pluripotency-associated gene activation without relying on exogenous transcription factor delivery.

## Simple Summary

Somatic cell reprogramming is an important technology for animal biotechnology, embryo engineering, and genetic improvement. However, the efficiency of reprogramming is limited by epigenetic barriers that restrict the activation of pluripotency-related genes. The long non-coding RNA *XIST* plays a central role in X-chromosome inactivation and contributes to epigenetic regulation during cell fate transitions. In this study, we investigated whether *XIST* knockdown could influence endogenous pluripotency-associated genes in IBRS-2 cells, a porcine female somatic cell line. Chemically modified antisense oligonucleotides (ASOs) were used to suppress *XIST* expression in IBRS-2 cells. *XIST* knockdown resulted in sustained increases in *KLF4* and *LIF* expression, together with transient activation of *NANOG* and other pluripotency-associated genes. KLF4 protein was detectable by antibody staining in a subset of ASO-2-treated cells. These findings indicate that *XIST* suppression can facilitate the partial activation of endogenous pluripotency-associated genes and may provide a potential molecular strategy for modulating cell reprogramming-associated gene expression in pigs.

## 1. Introduction

X-chromosome inactivation (XCI) is an essential epigenetic mechanism that equalizes X-linked gene dosage between male and female mammals [1]. The long non-coding RNA XIST plays a central role in this process by coating the inactive X chromosome (Xi) and recruiting chromatin-modifying complexes that establish transcriptional silencing [2]. Through coordinated histone modifications and DNA methylation, *XIST* establishes stable and heritable repression across the Xi [3]. *XIST* dynamically regulates the localization of macroH2A on Xi, contributing to the reversible regulation of X-chromosome silencing during female somatic cell reprogramming [4]. Additionally, the exogenous pluripotency-associated transcription factors (pTFs) promote formation of the active X-chromosome compartment (A-compartment) by repressing *XIST*, thus restoring biallelic X-chromosome activity in reprogramming [5]. X-chromosome reactivation (XCR) not only reverses the silencing state and restores transcriptional activity of the inactive X chromosome in reprogramming and early embryos [6,7], but also is closely associated with the acquisition of pluripotency [8]. XCI and XCR represent dynamically regulated developmental mechanisms essential for pluripotency regulation in embryonic stem cells (ESCs), induced pluripotent stem cells (iPSCs), and somatic cell nuclear transfer (SCNT) embryos [9].

Recent advances in cell reprogramming have enabled diverse strategies to induce pluripotency in somatic cells. Many conventional reprogramming approaches rely on the ectopic expression of pTFs [10,11,12,13]. *OCT4*, *SOX2* and *NANOG* can repress *XIST* transcript and promote X-linked gene reactivation during acquisition of pluripotency [14]. Furthermore, the previous studies demonstrated that combining the exogenous pTFs with *XIST* knockdown facilitated somatic cell reprogramming into iPSCs [4,15]. *XIST* knockdown rescues aberrant *XIST* expression on inactive X chromosome (Xi) in SCNT embryos, thereby enhancing X-linked gene reactivation and improving post-implantation survival by up to tenfold [16]. We found that microinjection of anti-*XIST* shRNA plasmids into porcine parthenogenetic embryos increased blastocyst cell numbers and upregulated the expression of endogenous *OCT4*, *SOX2*, and *NANOG* [17]. However, *XIST* knockdown alone appears insufficient to fully activate the endogenous pluripotency network during reprogramming [4,5,15]. Incomplete activation of endogenous pluripotency transcription factors following *XIST* knockdown may result from insufficient *XIST* repression. Therefore, improving the efficiency of *XIST* silencing may further alleviate *XIST*-mediated epigenetic barriers and facilitate the establishment of a stable endogenous pluripotency network during reprogramming.

Because *XIST* is a predominantly nuclear-localized lncRNA [18], *XIST* knockdown strategies should be more tailored to the subcellular localization characteristics. Because *XIST* is predominantly nuclear localized, knockdown strategies should be tailored to its subcellular localization, considering that RNAi-based approaches are generally more effective against cytoplasmic transcripts [4,16,17]. To further elucidate the effects of *XIST* knockdown on endogenous pTFs expression, we designed antisense oligonucleotides (ASOs) targeting the first exon of *XIST* based on previously reported anti-*XIST* shRNA sequences [17]. ASOs represent an effective strategy for targeting nuclear-localized lncRNAs. This is attributed to the predominant nuclear localization of RNase H, as well as the fact that ASOs modified with phosphorothioate (PS), 2′-O-methyl (2′-OMe), and locked nucleic acid (LNA) exhibit enhanced target affinity and nuclease stability [19,20].

IBRS-2 is a female-derived porcine kidney epithelial cell line that provides a reproducible and experimentally tractable somatic cell model. Because *XIST* is closely associated with X-chromosome inactivation in female cells, its female origin provides a relevant cellular context for investigating the potential effects of *XIST* depletion on endogenous pluripotency-associated gene expression. Previous work has characterized the expression and epigenetic regulation of porcine *XIST* in male and female cells and demonstrated that porcine *XIST* transcript is detected specifically in female cells [21]. Therefore, IBRS-2 cells were selected in the present study to investigate the temporal effects of *XIST* knockdown on endogenous pluripotency-associated transcription factors and related genes.

This study employed ASOs to silence *XIST* specifically in IBRS-2 cells. We demonstrate that *XIST* knockdown promotes the expression of endogenous pTFs, signaling genes and X-linked genes in IBRS-2 cells. These findings provide evidence that *XIST* knockdown may facilitate endogenous pTFs activation without introducing exogenous transcription factors or viral vectors in porcine female somatic cells.

## 2. Materials and Methods

### 2.1. Cell Culture

IBRS-2 cells, a porcine female somatic cell line, were obtained from Punosai Life Technology Co., Ltd. (Wuhan, China). Cells were cultured in Dulbecco’s Modified Eagle Medium (DMEM; Gibco, Grand Island, NY, USA) supplemented with 10% fetal bovine serum (FBS; Gibco BRL, Grand Island, NY, USA), 100 μg/mL penicillin, and 100 μg/mL streptomycin (penicillin-streptomycin; Gibco, Grand Island, NY, USA) at 37 °C in a humidified incubator containing 5% CO_2_. Cells were seeded in 24-well plates at a density of 1 × 10^5^ cells/mL, grown to 80–90% confluence, and transfected with anti-*XIST* shRNA plasmids and anti-*XIST* ASOs for 6 h using Lipofectamine^TM^ 3000 (Thermo Fisher Scientific, Carlsbad, CA, USA) according to the manufacturer’s instructions. The anti-*XIST* shRNA plasmids (p-shRNA2, p-shRNA5) used in this study were constructed in our laboratory and obtained from our previous work [17]. The anti-*XIST* ASOs (ASO-2, ASO-3, ASO-5, ASO-NC) were chemically synthesized by GenePharma (Shanghai, China). Their sequences are listed in Table 1.

### 2.2. Anti-XIST ASO Design and Synthesis

ASOs targeting the first exon of porcine *XIST* were designed based on previously published studies [17,20,22]. Specifically, the entire ASO backbone was modified with phosphorothioate (PS) linkages. Nucleotide modifications were introduced at both the 3′ and 5′ ends, with five modified nucleotides at each terminus—three with 2′-O-methyl (2′-OMe) modifications and two with locked nucleic acid (LNA) modifications. All ASOs were labeled with FAM at the 3′ terminus for fluorescence tracking. All oligonucleotides were synthesized by GenePharma (Shanghai, China) and diluted to 20 μM in RNase-free water. The sequences of all ASOs are listed in Table 1.

### 2.3. XIST Knockdown

The anti-*XIST* shRNA plasmids and anti-*XIST* ASOs were respectively diluted to final concentrations of 80 μg/mL and 20 μM in DEPC-treated water. A mixture containing 0.8 μg plasmid or 2.5 μL ASO solution and 50 μL Opti-MEM was prepared and incubated. Meanwhile, 1.5 μL Lipofectamine^TM^ 3000 and 1 μL P3000 reagent were diluted in another 50 μL Opti-MEM following the same protocol. The two mixtures were combined, incubated for 15 min at room temperature, and then added to each well pre-filled with 400 μL Opti-MEM. After 6 h incubation at 37 °C with 5% CO_2_, the medium was replaced with complete medium containing 5% FBS, and the cells were continuously cultured under the same conditions for subsequent experiments.

### 2.4. RT-qPCR Detection

Total RNA was extracted from IBRS-2 cells using TRIzol^TM^ reagent (Invitrogen, Carlsbad, CA, USA) according to the manufacturer’s protocol. DNase-treated RNA (1 μg) was reverse-transcribed into cDNA in a 20 μL reaction using the PrimeScript^TM^ II First-Strand cDNA Synthesis Kit (TaKaRa, Dalian, China) at 42 °C for 15 min. Quantitative PCR was performed using SYBR Green qPCR Master Mix-Plus (TOYOBO, Osaka, Japan) in a 20 μL reaction on a SLAN-965 Real-Time PCR System (Hongshi, China). Relative gene expression was normalized to 18S rRNA using the 2^−ΔΔCt^ method. Thermal cycling conditions were as follows: initial denaturation at 95 °C for 1 min, followed by 40 cycles of 95 °C for 15 s, 58 °C for 30 s, and 72 °C for 15 s. Primer sequences are listed in Table 2.

### 2.5. Immunofluorescence Staining

IBRS-2 cells were fixed with 4% (*w*/*v*) paraformaldehyde for 40 min, followed by permeabilization with 0.5% (*v*/*v*) Triton X-100 in DPBS for 60 min at 25 °C. Samples were washed three times with DPBS and then blocked with 3% (*w*/*v*) BSA in DPBS for 2 h at 25 °C. Cells were incubated overnight at 4 °C with primary antibodies against *KLF4* (AF2506; Beyotime, Shanghai, China) at a 1:100 dilution. After three washes, samples were incubated with Alexa Fluor^®^ 555–conjugated donkey anti-rabbit IgG (A0453, Beyotime, China) at a 1:500 dilution for 1 h at room temperature. Samples were washed again, stained with 4,6-diamidino-2-phenylindole (DAPI), and observed using a fluorescence microscope (Nikon Corp., Tokyo, Japan).

### 2.6. Statistical Analysis

Statistical differences among groups were analyzed by one-way analysis of variance (ANOVA) followed by Tukey’s multiple comparison test. Data are presented as the mean ± standard deviation (SD). Differences were considered statistically significant at *p* < 0.05 and at *p* < 0.001. All experiments were performed in biological triplicate.

## 3. Results

### 3.1. Screening of ASO Targeting XIST

**Figure 1:**
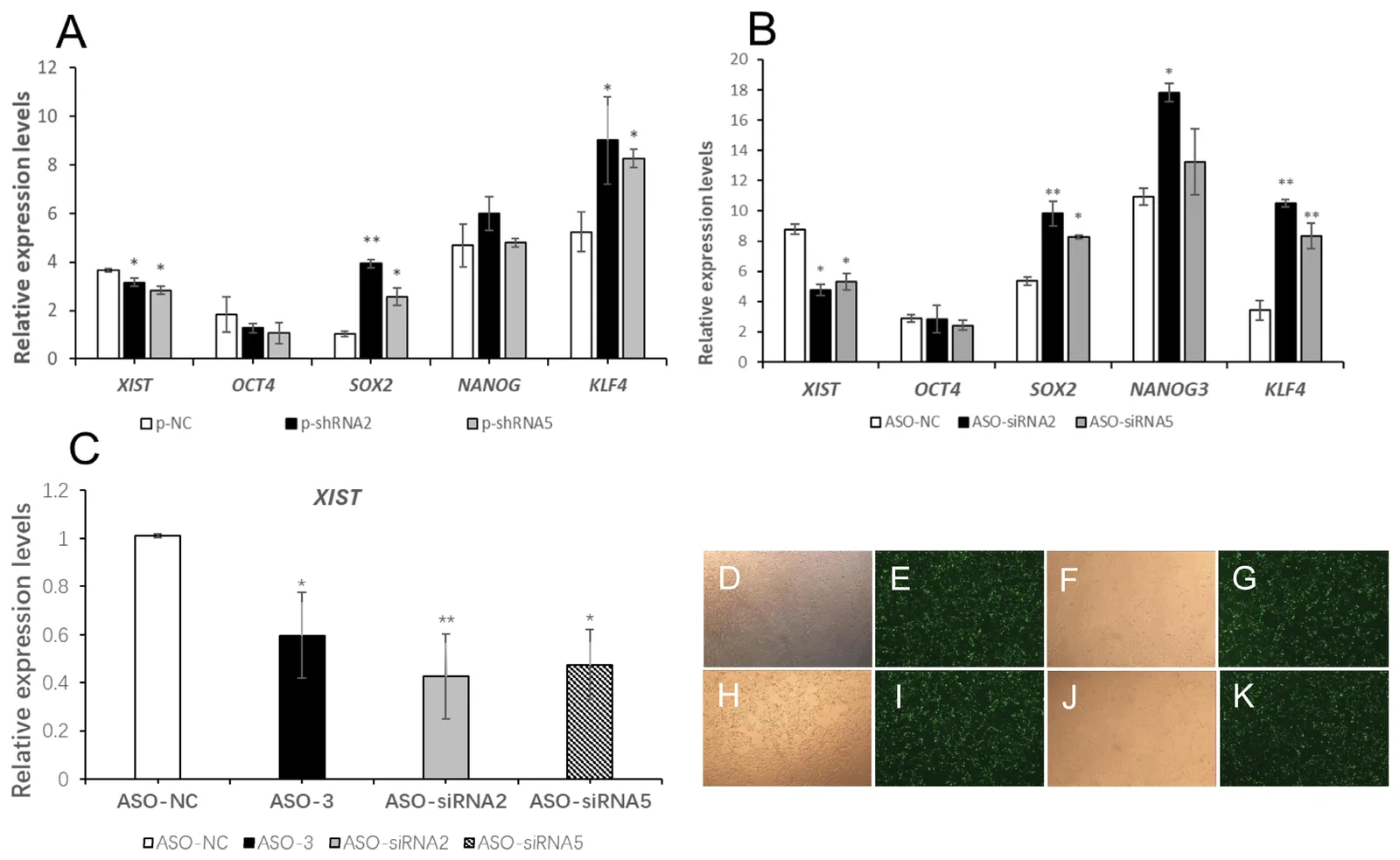
Effects of anti-XIST ASOs on transcript levels of endogenous pTFs in IBRS-2 cells. (**A**) anti-*XIST* shRNA plasmid influenced the exogenous pTFs transcript levels. (**B**) anti-*XIST* ASO knockdown *XIST* expression. (**C**) anti-*XIST* ASO influenced the endogenous pTFs transcript levels. (**D**,**E**) Control group (ASO-NC). (**F**,**G**) Treatment Group ASO-3. (**H**,**I**) Treatment Group (ASO-2). (**J**,**K**) Treatment Group (ASO-5). (**D**,**F**,**H**,**J**) Bright-field image. (**E**,**G**,**I**,**K**) FAM-labeled green fluorescence image. Original magnification: 40×. The relative expression levels were determined by Q-PCR at 24 h after transfection and normalized to 18S rRNA. * *p* < 0.05 and ** *p* < 0.001.

IBRS-2 cells were transfected with anti-XIST shRNA plasmids and anti-XIST ASOs. The transcript levels of *XIST* and pTFs (*OCT4*, *SOX2, NANOG*, and *KLF4*) were analyzed 24 h post-transfection. *XIST* transcript levels were significantly reduced in both the p-shRNA2 and p-shRNA5 groups, along with significant upregulation of *KLF4* and *SOX2*. However, *NANOG* and *OCT4* transcript levels showed no significant changes in either group (Figure 1A). To further enhance the expression efficiency of the endogenous pTFs, the shRNAs were used as a preliminary reference for target-site selection, whereas ASOs were subsequently screened for efficient XIST knockdown. Four anti-XIST ASOs were designed and synthesized, followed by transfection into IBRS-2 cells. Fluorescence microscopy at 24 h post-transfection revealed comparable transfection efficiencies among the ASO-treated groups (Figure 1D–K). Quantitative PCR (qPCR) confirmed that three ASOs significantly reduced *XIST* expression, with ASO-2 producing the strongest knockdown effect (Figure 1B). At 24 h post-transfection, *OCT4* transcript level showed no significant change, whereas those of *SOX2*, *NANOG*, and *KLF4* were significantly upregulated (Figure 1C).

### 3.2. XIST Knockdown Influenced the Transcript Levels of the Pluripotency-Associated Genes

**Figure 2:**
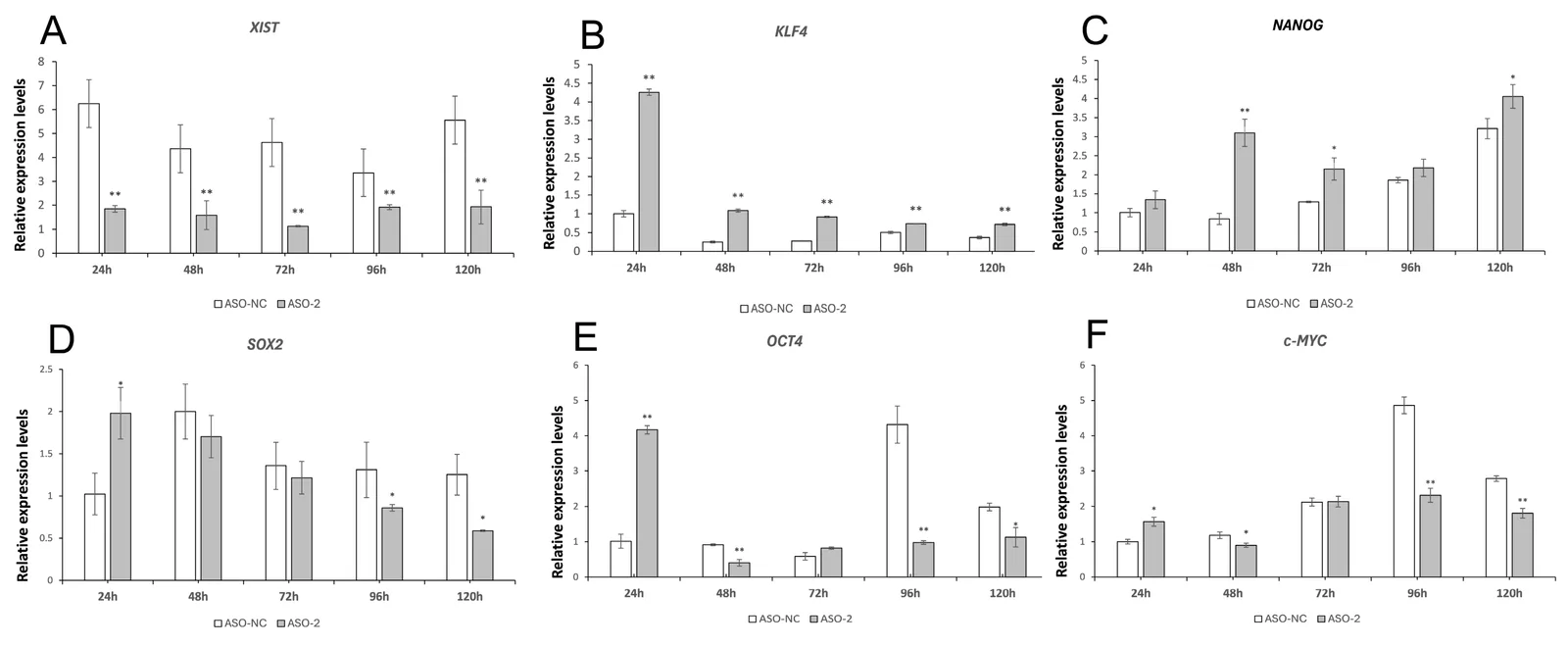
Effects of *XIST* knockdown on transcript levels of the pTFs in IBRS-2. (**A**) Relative transcript level of *XIST*. (**B**) Relative transcript level of *KLF4*. (**C**) Relative transcript level of *NANOG*. (**D**) Relative transcript level of *SOX2*. (**E**) Relative transcript level of *OCT4*. (**F**) Relative transcript levels of *c-MYC*. The relative expression levels of the six genes were determined by Q-PCR at 24 h, 48 h, 72 h, 96 h, 120 h after transfection of ASO-2 and normalized to 18S rRNA. For adjacent pairs of columns, * *p* < 0.05 and ** *p* < 0.001.

To determine whether *XIST* knockdown continuously affects the expression of endogenous pTFs, the transcript levels of *OCT4*, *c-MYC*, *KLF4*, *NANOG*, and *SOX2* were analyzed in IBRS-2 cells from 24 h to 120 h post-transfection. *XIST* expression was significantly suppressed throughout this period (Figure 2A), whereas *KLF4* expression was markedly upregulated (Figure 2B). *SOX2*, *OCT4*, and *c-MYC* transcript levels increased significantly at 24 h post-transfection but were significantly downregulated at 96 and 120 h post-transfection (Figure 2D–F). In contrast, *NANOG* remained significantly upregulated at 48 h, 72 h, and 120 h post-transfection (Figure 2C).

**Figure 3:**
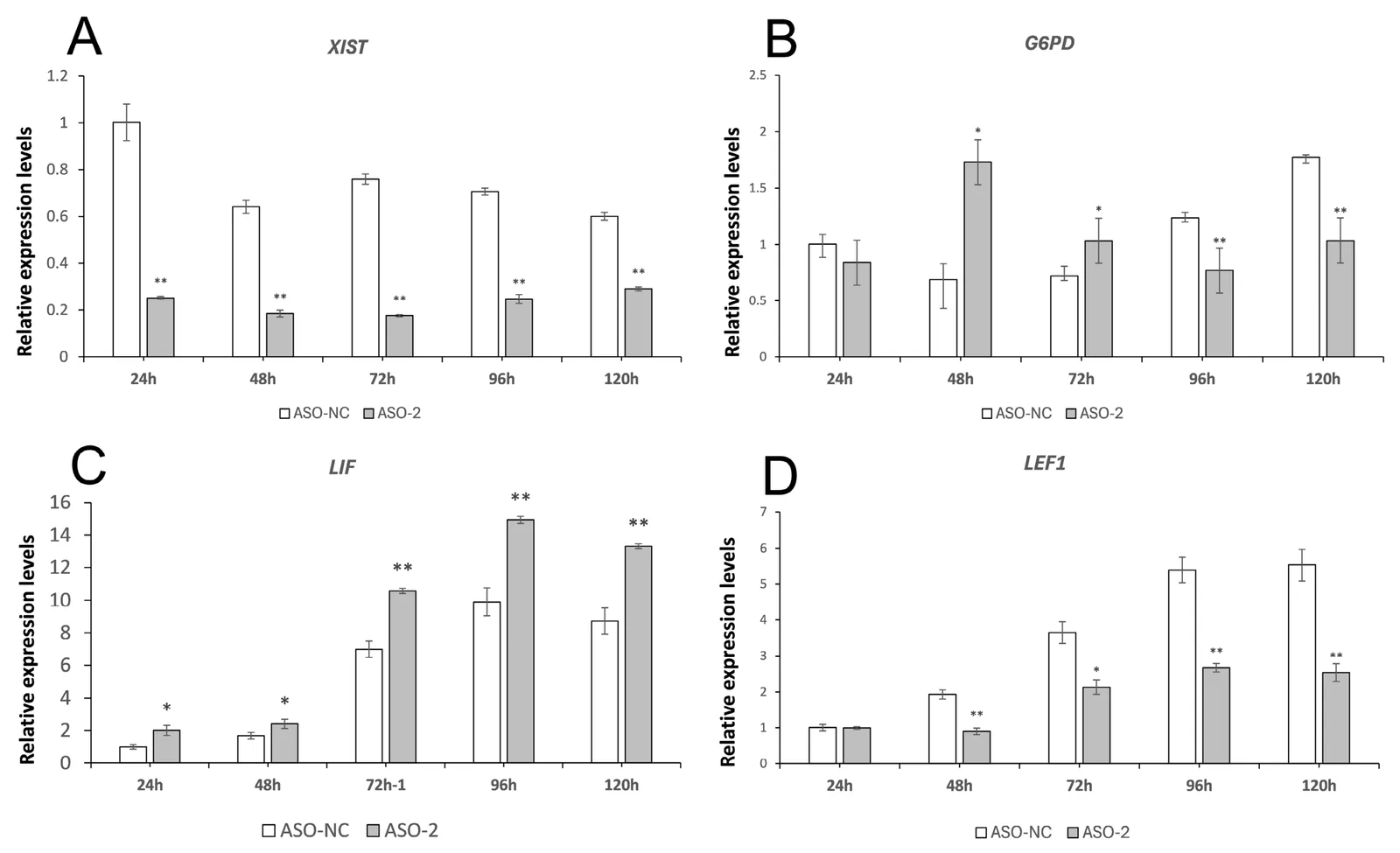
Effects of *XIST* knockdown on transcript levels of *LIF*, *LEF1* and *G6PD* in IBRS-2 cells. (**A**) Relative transcript level of *XIST*. (**B**) Relative transcript level of *G6PD*. (**C**) Relative transcript level of *LIF*. (**D**) Relative transcript level of *LEF1.* The relative expression levels of the four genes in IBRS-2 from 24 h to 120 h after transfection of ASO-2 (gray histogram) and negative control (blank histogram). The relative transcript levels of the four genes were respectively determined by Q-PCR at 24 h, 48 h, 72 h, 96 h, 120 h after transfection and normalized to 18S rRNA. For adjacent pairs of columns, * *p* < 0.05 and ** *p* < 0.001.

To further investigate whether *XIST* knockdown influences signaling genes and X-linked genes, *LIF*, *LEF1*, and *G6PD* transcript levels were analyzed in IBRS-2 cells from 24 h to 120 h post-transfection. *XIST* expression was significantly reduced throughout this period (Figure 3A), while *LIF* expression exhibited sustained upregulation (Figure 3C). *LEF1* expression progressively decreased beginning at 48 h, reaching significant suppression between 48 h and 120 h (Figure 3D). The X-linked gene *G6PD* displayed transient activation at 48 h and 72 h post-transfection, followed by marked downregulation at 96 h and 120 h post-transfection (Figure 3B).

### 3.3. XIST Knockdown Influenced KLF4 Expression

**Figure 4:**
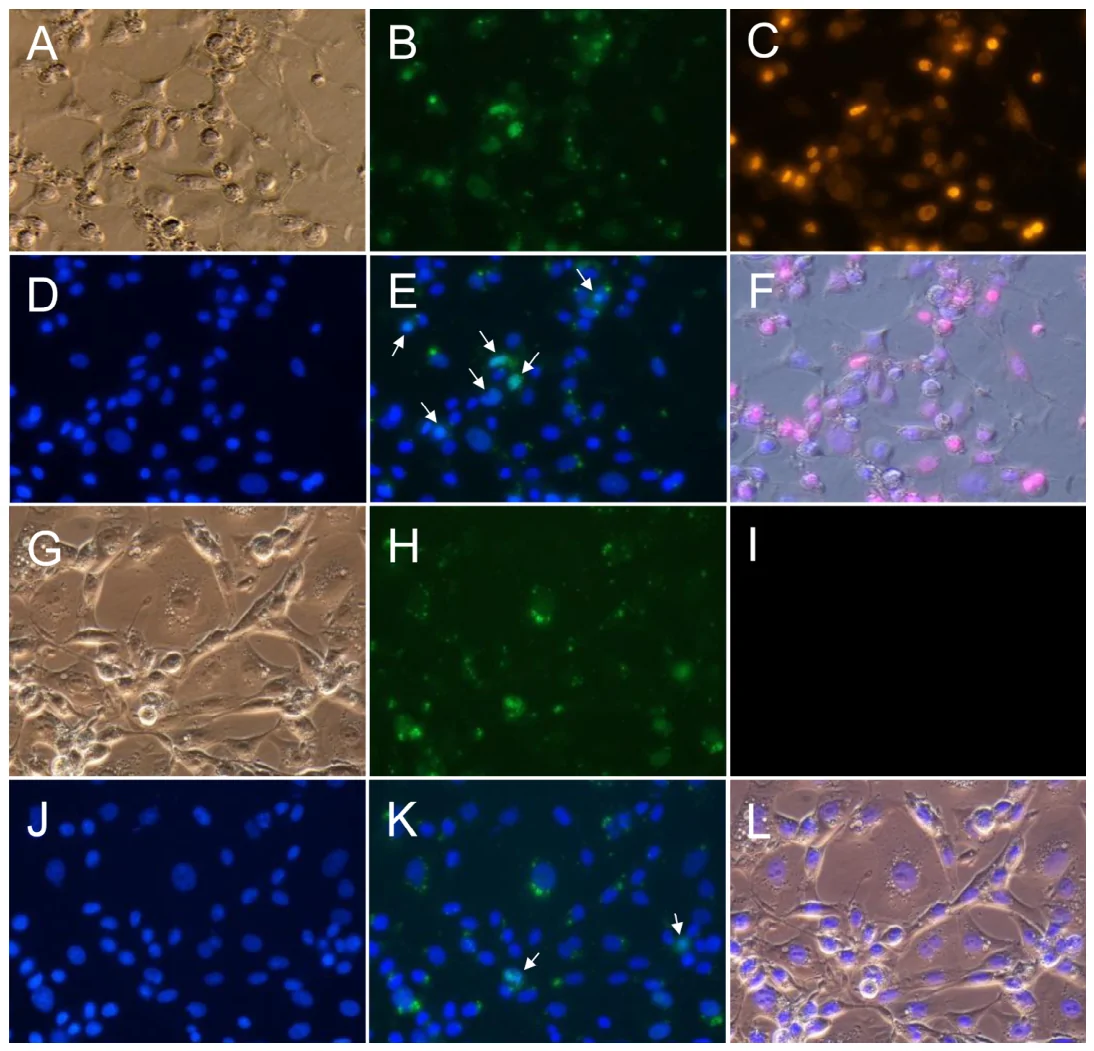
Fluorescent staining of IBRS-2 at 120 h post-transfection with ASO-2. (**A**–**F**) Treatment Group (ASO-2). (**G**–**L**) Control group (ASO-NC). (**A**,**G**) Bright-field image of IBRS-2. (**B**,**H**) FAM-labeled green fluorescence image. (**C**,**I**) Red immunofluorescence image of KLF4. (**D**,**J**) Blue fluorescence image (nuclei are counterstained with DAPI). (**E**,**K**) Merged image of green fluorescence and blue fluorescence (the white arrows point to cyan). (**F**) Merged image of red immunofluorescence, blue fluorescence and bright-field image (KLF4-positive cells display pink staining). (**L**) Merged image of red immunofluorescence, blue fluorescence and bright-field image (KLF4-negative cells appear blue). Original magnification: 400×.

Following ASO-2-mediated *XIST* knockdown, sustained suppression of *XIST* expression was accompanied by time-dependent changes in pluripotency-associated genes. Notably, *KLF4* transcript level was consistently and significantly upregulated from 24 h to 120 h in IBRS-2. At 120 h post-transfection, immunofluorescence and DAPI staining revealed KLF4 expression in a subset of cells (Figure 4C,F). FAM-labeled ASO-2 was widely distributed within cells (Figure 4B,H), with partial nuclear localization observed (Figure 4E,K). The nuclear-associated FAM signal indicates that ASO-2 was detectable in the nucleus at 120 h post-transfection (Figure 4E,K).

## 4. Discussion

Although LncRNAs can be nuclear, cytoplasmic, or dual-localized [27], *XIST* RNA is specifically localized to the inactive chromosome within the nucleus of female cells [28]. ASO and siRNA represent distinct gene-silencing modalities that differ in mechanisms of action, delivery strategies, and efficiency when targeting lncRNAs. In the present study, two strategies were used to interfere with *XIST* expression. Although anti-*XIST* shRNA plasmid treatment significantly reduced *XIST* expression, endogenous *OCT4* and *NANOG* expression did not return to previous levels [17]. In contrast, *XIST*-targeting ASO-2 achieved sustained *XIST* suppression for up to 120 h in IBRS-2 cells, accompanied by transient activation of *SOX2*, *NANOG*, and *KLF4* at 24 h post-transfection. These results suggest that ASO-mediated *XIST* silencing provides an efficient approach for targeting nuclear lncRNAs [19]. Because the experiments were primarily performed using a single ASO-2 sequence, potential sequence-dependent or off-target effects cannot be completely excluded.

As the principal regulator of XCI, *XIST* coordinates transcriptional silencing of the inactive X chromosome (Xi) through its RNA scaffold, recruiting more than 100 chromatin-associated proteins to establish stable epigenetic repression [29]. Furthermore, *XIST* regulates cellular substructures by recruiting regulatory elements, functional proteins, and transcription factors to facilitate chromatin remodeling and subcellular structural organization [4]. Following *XIST* knockdown, the reduced *XIST* RNA abundance may weaken the recruitment or retention of chromatin modifiers, DNA methyltransferases, transcription factors, and RNA-binding proteins (RBPs) at the Xi. This may alter the transcriptional state of genomic regions on the Xi [30,31] and potentially contribute to the observed changes in pTF and X-linked gene expression. The delocalization of *XIST* from the Xi is considered an important event associated with X-linked gene reactivation during XCR [31,32,33,34]. Most X-linked gene reactivation events occur concomitantly with *XIST* silencing and re-expression of endogenous pluripotency factors [5,35]. However, in the present study, *XIST* knockdown did not result in sustained activation of *G6PD* transcription, as *G6PD* expression increased transiently at 48 and 72 h but decreased thereafter. Upon 120 h *XIST* knockdown, the expression profile of *G6PD* in somatic cells was comparable to that of *FOXP3* in porcine embryos receiving the same perturbation [17].

*XIST* plays a crucial regulatory role in somatic cell pluripotency. Its first intron directly interacts with *NANOG*, *OCT4*, and *SOX2* to recruit repressive factors that silence the *XIST* transcript, thereby modulating pluripotency through XCR [36]. Through *XIST* knockdown, X-chromosome activity becomes closely coordinated with pTFs expression. Moreover, *OCT4*, *SOX2*, *KLF4*, and *c-MYC* have been implicated in coordinating XCR and pluripotency acquisition [36,37] and trigger XCR during the reprogramming of female somatic cells into iPSCs [38]. However, *XIST* repression is required at later stages of iPSC induction [4,39,40,41,42]. In this study, *SOX2*, *OCT4*, and *c-MYC* did not show significant upregulation at 96 h–120 h post-transfection, contrasting with prior findings in porcine *XIST* knockdown embryos [17]. This discrepancy may result from natural epigenetic resetting in blastocyst cells, where the transition from totipotency to pluripotency is inherently more efficient. Activation of *KLF4* and *NANOG* following *XIST* suppression suggests that *XIST* knockdown may contribute to the activation of pluripotency-associated genes. *KLF4* is one of the core transcription factors involved in somatic cell reprogramming and plays an important role in the establishment of pluripotency [43,44]. In the present study, *KLF4* expression remained significantly elevated following *XIST* knockdown, suggesting that *XIST* may participate in the regulation of endogenous pluripotency-associated transcriptional programs. *KLF4*-centered chromatin remodeling is associated with enhancer rewiring and activation of target gene transcription [43]. Furthermore, epiblast-derived pluripotent stem cells can be reprogrammed into naïve pluripotent cells through *KLF4* transfection [44]. However, *KLF4* and *NANOG* activation induced by *XIST* knockdown alone was insufficient to achieve complete lineage reprogramming, indicating that successful reprogramming likely requires *XIST* knockdown in combination with exogenous pTFs or small-molecule inhibitors [4,38].

*LIF* regulates cellular epigenetic remodeling through complex interactions with other signaling pathways, enhancing responsiveness to reprogramming factors and promoting the transition to a pluripotent state [45]. In combination with *OCT4*, *SOX2*, *KLF4*, and *c-MYC*, *LIF* improves reprogramming efficiency and stabilizes the epigenetic plasticity of partially reprogrammed intermediates [46]. LIF also collaborates with inhibitors of the kinases GSK3/MEK, mediating mitochondrial metabolism via the LIF-Stat3 signaling axis to induce genomic hypomethylation [47]. This metabolic regulation is considered important for the initiation of cell reprogramming and may contribute to XCR [45]. In this study, *XIST*-targeting ASO treatment induced sustained activation of endogenous *LIF* transcript while suppressing *LEF1* expression. *LEF1* binds the promoter regions of *c-MYC*, *OCT4*, and *NANOG* to maintain pluripotency [48]. During fibroblast dedifferentiation, *LEF1* promotes differentiation arrest by activating *NANOG* and *OCT4* transcription [49].

Previous studies have shown that inhibition of Wnt/β-catenin signaling and downregulation of *LEF1* can reduce *OCT4* and *NANOG* expression during embryonic stem cell differentiation [48,50,51]. In the present study, *XIST* knockdown resulted in sustained suppression of *LEF1* expression from 48 to 120 h post-transfection, whereas *OCT4* and *SOX2* expression increased transiently at 24 h and subsequently declined. Similarly, *c-MYC* showed a transient increase at 24 h followed by a decrease at later time points. The temporal association between *LEF1* suppression and the subsequent decline of *OCT4*, *SOX2*, and *c-MYC* suggests that altered Wnt/LEF1 signaling may be involved in the dynamic regulation of pluripotency-associated gene expression following *XIST* knockdown. However, Wnt/LEF1 pathway activity was not directly assessed in the present study, and a causal relationship between *LEF1* suppression and the later decline of these factors cannot be established. Further studies are therefore required to determine whether Wnt/LEF1 signaling contributes to this temporal regulatory pattern.

Several limitations should be considered when interpreting the present findings. First, the experiments were performed using the immortalized female-derived IBRS-2 cell line rather than primary porcine somatic cells. Therefore, whether the temporal expression responses observed following *XIST* knockdown are conserved across different porcine somatic cell types remains to be determined. Second, a sex-matched male counterpart of IBRS-2 was not included in the present study. Although male porcine kidney-derived cell lines are available, their different cellular and genetic backgrounds would prevent a direct interpretation of sex-specific effects. Thus, whether the observed responses are strictly female-specific cannot be established from the current experimental design. In addition, although multiple anti-*XIST* ASO candidates were initially screened, the subsequent time-course experiments primarily relied on ASO-2; therefore, sequence-dependent or off-target effects cannot be completely excluded. Future studies using primary porcine somatic cells, independent *XIST*-targeting sequences, and sex-matched cellular models will be valuable for further validating and extending these observations.

## 5. Conclusions

In summary, *XIST* knockdown via ASO-2 activated a subset of endogenous pTFs in IBRS-2 cells. Sustained upregulation of endogenous *KLF4* and *LIF*, phased activation of *NANOG*, *OCT4*, *SOX2*, *c-MYC*, and the X-linked gene G6PD were observed following *XIST* suppression. These findings indicate that *XIST* knockdown can partially modulate the endogenous pluripotency-associated genes in porcine female somatic cells, with gene-specific and time-dependent responses. However, *XIST* knockdown alone was insufficient to induce a coordinated and sustained activation of the endogenous pluripotency network, and whether these transcriptional changes contribute to functional somatic cell reprogramming remains to be determined.

## Figures and Tables

**Table 1:** Sequences of ASOs and shRNAs targeting the porcine *XIST*.

Groups	Sequence of Nucleotide (5′ → 3′)	Targets and Positions in the Template #
shRNA2	GCCTTGAGTGATTATTATG CGGAATTCACTGATGATAC	2192-GCCTTAAGTGACTACTATG-2210
shRNA5	GGTGCTTGGGATTGTTGAT CCATGAACCCTGACAATTA	11967-GGTACTTGGGACTGTTAAT-11985
ASO-2	C*A*U*A*G*t*a*g*t*c*a*c*t*t*A*A*G*G*C	2192-GCCTTAAGTGACTACTATG-2210
ASO-3	U*U*G*A*A*c*c*c*t*g*c*g*c*c*t*A*A*A*C*C	1145-GGTTTAGGCGCAGGGTTCAA-1164
ASO-5	A*U*U*A*A*g*a*g*t*c*c*c*a*a*G*U*A*G*C	11967-GGTACTTGGGACTGTTAAT-11985
ASO-NC	G*C*G*U*A*t*t*a*t*a*g*c*c*g*a*T*T*A*A*C	No target sequence

p-shRNA2 and ASO-2 share one target site on the KC753465.1 sequence, as do p-shRNA5 and ASO-5 for another site; lowercase letter: DNA; capital letter: RNA; *: PS linkages; single underline: 2′-OMe (2′-O-methyl); double underline: LNA (locked nucleic acid); #: The 1-17184 bases of the KC753465.1 sequence were used as the template for ASO designing.

**Table 2:** Primers for Q-PCR Analysis.

Gene	Primer Sequence (5′ → 3′)	Annealing (C)	Size (bp)	Reference/Accession Numbers
*XIST*	F: GCTCCAACCAATCTAAAAGGA R: ATGCCCCATCTCCACCTAA	58.4	131	[23]
*SOX2*	F: AACCAGAAGAACAGCCCAGAC R: TCCGACAAAAGTTTCCACTCG	58.4	155	[24]
*NANOG*	F: CATCTGCTGAGACCCTCGAC R: GGGCTTGTGGAAGAATCAGG	58.4	181	[25]
*OCT4*	F: GAAGGTGTTCAGCCAAACGAC R: CGATACTTGTCCGCTTTC	58.4	185	[24]
*c-MYC*	F: CGGACACGGAGGAGAATG R: TGCTTGGACAGACAGGATG	58.4	182	NM_001005154.1
*KLF4*	F: TGGGTGCGGAGGAACTGCTA R: GGGACTGACCTTGGTAATGGAGC	58.4	153	NM_001031782.2
*LIF*	F: CACTGGAAACACGGGGCA R: AGGGCGGGAAGTTGGTCA	54	229	[26]
*LEF1*	F: GCTATTGTAACGCCTCAG R: CTCTTGCTCCTTTCTCTG	54	96	NM_001129967.1
*G6PD*	F: CCTACGGCAACAGATACAAGAA R: AGCAGCGGCGTGAAGATG	54	139	XM_021080744.1
*18srRNA*	F: GCCCGAAGCGTTTACTTTGA R: CCGCGGTCCTATTCCATTATT	54 or 58.4	93	NR_002170.3

F: forward primer; R: reverse primer.

## Data Availability

The data presented in this study are available on request from the corresponding authors.
